# Oral Health Knowledge, Oral Health–Seeking Behavior, and Associated Factors Among the Rural Population of Ernakulam District: A Cross-Sectional Study

**DOI:** 10.7759/cureus.99666

**Published:** 2025-12-19

**Authors:** Das P Anaswara, Sobha George

**Affiliations:** 1 Public Health, Amrita Institute of Medical Sciences and Research Center, Kochi, IND; 2 Community Medicine, Amrita Institute of Medical Sciences and Research Center, Kochi, IND

**Keywords:** health equity, health-seeking behavior, kerala, oral health literacy, rural health

## Abstract

Introduction: Oral diseases affect over 3.5 billion people globally, with a disproportionate burden in low- and middle-income countries such as India. Oral health knowledge (OHK) strongly influences oral health-seeking behavior (OHSB), yet data from rural Kerala are scarce. This study aims to assess OHK and OHSB, examine their association, and identify their determinants among rural adults in Ernakulam district.

Materials and methods: A community-based cross-sectional study was conducted among 160 adults aged 18-60 years in Njarakkal Panchayat, Kerala, using multistage cluster sampling. Data were collected through a pretested, validated interview schedule assessing OHK (37 items) and OHSB (7 items). Chi-square tests and multivariable log-binomial regression were used to identify predictors of adequate OHK and positive OHSB.

Results: Adequate OHK and OHSB were found in 48.12% (95% confidence interval (CI): 38.58-57.80) and 41.25% (95% CI: 32.15-51.10) of participants, respectively. Younger age (<45 years) (adjusted odds ratio (AOR) (CI) (1.46 (1.15-2.12)), higher education (AOR (CI) (5.31 (3.97-6.66)), employment (AOR (CI) (1.69 (0.97-2.40)), and above-poverty-line status (AOR (CI) (3.30 (2.41-4.19)) were significantly associated with adequate OHK. Adequate OHK (AOR (CI) (3.35 (2.46-4.25)), younger age (AOR (CI) (1.27 (1.04-1.54)), higher education (AOR (CI) (4.84 (3.34-6.33)), and higher socioeconomic status(AOR (CI) (2.34 (1.54-3.13)) independently predicted positive OHSB. Tooth pain was the most common reason for dental visits (61.3%), while 72.9% avoided care, perceiving mild issues as self-manageable. Nearly half preferred private clinics due to poor accessibility of government services.

Conclusion: OHK emerged as a key predictor of OHSB in rural Kerala. Strengthening public dental services, integrating oral health education into primary care, and developing community-based programs can improve preventive practices, reduce delays in care-seeking, and narrow rural-urban disparities.

## Introduction

Oral health, as defined by the World Health Organization (WHO), is “the state of the mouth, teeth, and orofacial structures that enables essential functions such as eating, breathing, and speaking, and includes psychosocial aspects like self-confidence and well-being” [1]. Globally, oral diseases remain among the most prevalent non-communicable conditions. The 2016 Global Burden of Disease Study estimated that dental caries and periodontal diseases affect 3.58 billion people worldwide, including 2.4 billion adults and 486 million children [2-4]. These conditions pose a major public health challenge not only in high-income but also in low- and middle-income countries [5], with established links to systemic diseases such as cardiovascular disorders and diabetes, owing to shared risk factors and bidirectional relationships [6].

Oral hygiene is shaped by both nonmodifiable factors, such as age, gender, education, and socioeconomic status, and modifiable behaviors, including diet, tobacco use, and alcohol consumption [7,8]. With rising life expectancy, maintaining good oral health has become increasingly important for quality of life, and the WHO has identified oral disease prevention and health promotion as global priorities [9].

In India, poor oral health awareness, limited rural dental infrastructure, and reliance on costly private services hinder oral health-seeking behavior (OHSB), exacerbated by social disparities in income and education. Despite this knowledge, oral health has received limited policy attention in the past two decades [5,8,10-12]. Nearly 95% of Indians experience dental problems [6], with caries and periodontal diseases impairing daily functioning and quality of life, even when not life-threatening. Moreover, India’s high consumption of smoked and smokeless tobacco has made it the “oral cancer capital of the world,” disproportionately affecting rural and socioeconomically disadvantaged populations [1,5].

Kerala, though performing strongly on most health indicators, continues to lag in oral health literacy, access, and service utilization. Dental caries affects 37-69% of the population, periodontal diseases 65-78%, and the incidence of oral cancer per lakh population is 16.4-21.6 in males and 6.4-9.1 in females [13,14]. Public dental services are limited, forcing many residents to depend on distant tertiary centers or expensive private facilities, thereby widening rural-urban inequities.

Oral health literacy, defined as “the degree to which individuals can obtain, process, and understand basic oral health information and services needed to make appropriate health decisions,” is a key determinant of health-seeking behavior (HSB) and service utilization [15,16]. Low oral health literacy reduces preventive practices, impairs communication between patients and providers, and increases the risk of poor oral hygiene [17]. HSB, which refers to the actions individuals undertake to maintain or restore health, is closely linked to oral health knowledge (OHK) as well as to accessibility of care [7,17].

Previous Indian studies have typically examined OHK or HSB in isolation, without evaluating both dimensions together across a broad adult age range. Evidence from rural communities, where disparities in literacy, access, and affordability are most pronounced, remains scarce [4,8-10,18]. Therefore, the primary objective of this study was to assess OHK and OHSB among adults in a rural coastal Panchayat of Ernakulam district, and the secondary objectives were to examine the association between OHK and OHSB and to identify sociodemographic factors associated with both outcomes.

## Materials and methods

This community-based cross-sectional study was conducted to assess OHK, OHSB, and their associated factors among adults. Data were collected through a structured, interview-based survey in Njarakkal Panchayat, Ernakulam district, Kerala. Although only one Panchayat was included, it was randomly selected from the 82 Panchayats in the district, ensuring an unbiased selection process. According to the Census 2011, Njarakkal Panchayat in Ernakulam had a high literacy rate (97.46%), which is comparable to the average rural literacy rate of Ernakulam district (95.18%). This similarity in educational attainment suggests that Njarakkal shares a community profile consistent with other rural Panchayats in the region. Although Panchayat-level poverty statistics are not publicly available, Njarakkal and neighboring coastal Panchayats such as Chellanam exhibit comparable socioeconomic characteristics, being predominantly dependent on coastal rural livelihoods, including fishing, agriculture, small-scale informal labor, and daily-wage occupations. These shared demographic and occupational features provide support for the appropriateness of Njarakkal as a representative coastal rural Panchayat for this study. The Panchayat, a local self-governed administrative unit, comprises 16 wards that served as clusters for sampling. Data collection was carried out between August and October 2023.

Participants were included in the study if they were adults aged 18-60 years and had been permanent residents of Njarakkal Panchayat for at least one year. Individuals were excluded if they had physical disabilities that limited their ability to participate in the survey or if they had mental disabilities or cognitive impairment that could interfere with understanding the questions or providing reliable responses.

The required sample size was estimated using the formula: N = Z² × P(1-P)/d², where P = prevalence of adequate OHK (assumed 20% based on a previous study [2]), d = absolute precision (9%), and Z = 1.96 at a 95% confidence level. The calculated minimum sample size was 75. After adjusting for the cluster sampling design, a design effect of 2 was applied, giving an adjusted sample size of 150.

However, because the study used multistage cluster sampling with 16 wards serving as clusters, the sample size was rounded up to 160 to allow selection of an equal number of participants from each cluster. This ensured uniform representation across wards and avoided unequal cluster weights. Thus, 10 participants were selected from each of the 16 clusters, yielding a final sample size of 160.

**Figure 1:**
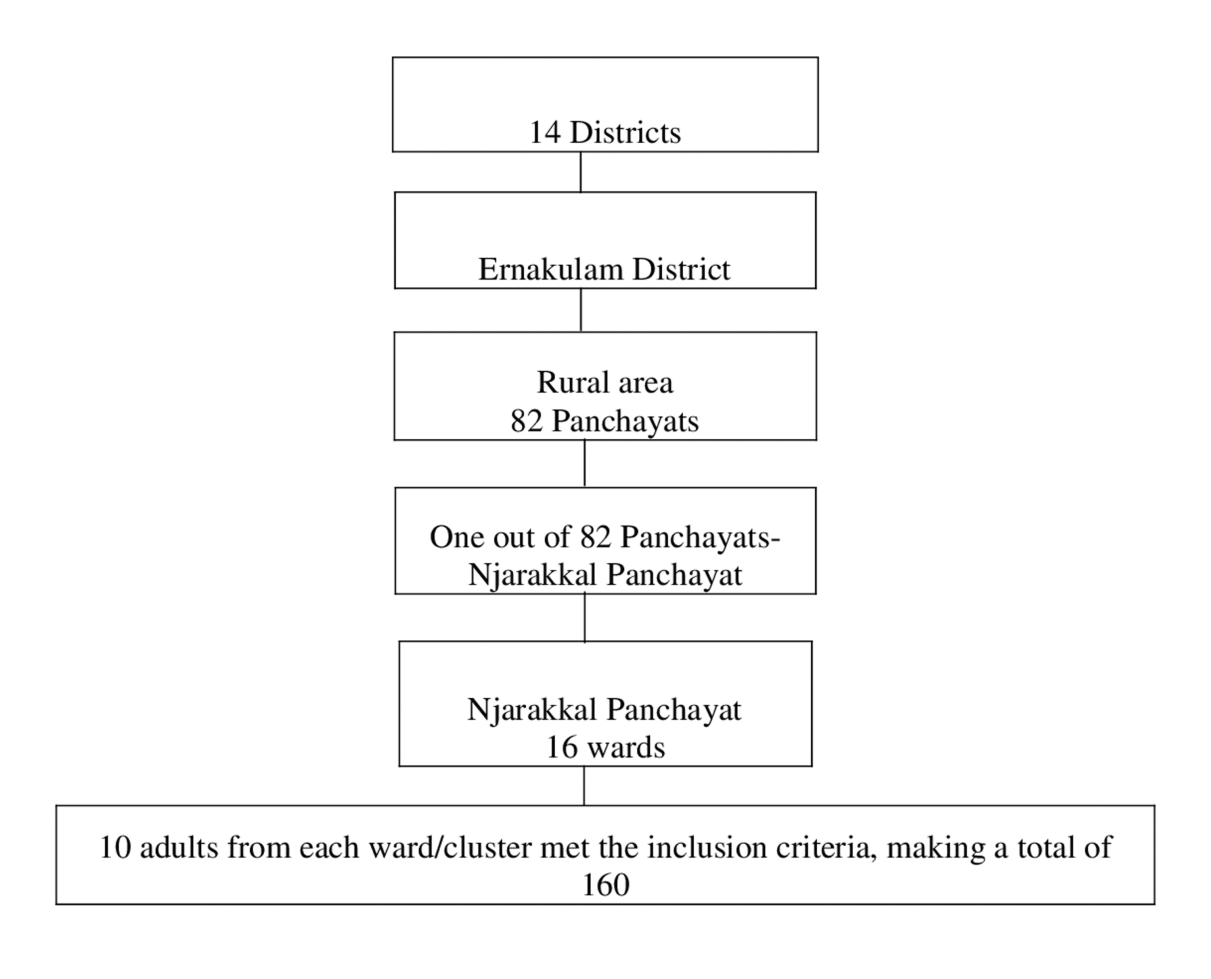
Sample selection process

Sampling followed a multistage cluster approach. One Panchayat was randomly selected from the 82 Panchayats in Ernakulam district. All 16 wards of the selected Panchayat were included as clusters. Ten participants were randomly chosen from each ward, with only one participant recruited per household to avoid clustering bias (Figure 1).

A total of 172 households were approached, and 160 individuals consented, giving a response rate of 93.0%. Data were collected using a structured questionnaire comprising 37 OHK items and seven OHSB items. The instrument was adapted from previously validated oral health KAP tools [9,10,18-20] and contextualized for the local culture and language. Content and face validity were assessed qualitatively by a panel of three public health and three dental experts, through independent review and consensus regarding item relevance, clarity, and appropriateness. No quantitative validity indices were calculated. Necessary modifications were made before final administration. The questionnaire was pretested among 20 adults from a demographically similar area to ensure clarity, cultural appropriateness, and comprehensibility.

For this study, OHK and OHSB were operationally defined as the total number of correct or positive responses to their respective questionnaire items. Each item was scored as “1” for a correct/positive response and “0” otherwise. Total OHK and OHSB scores were calculated and dichotomized using the sample mean as the cut-off. Scores above the mean were categorized as “adequate OHK” or “positive OHSB,” while scores at or below the mean were classified as “inadequate OHK” or “negative OHSB” [21].

All interviews were conducted face-to-face in Malayalam by a single trained investigator using the validated questionnaire to ensure uniform delivery. Each interview followed the same sequence, demographics, OHK items, and OHSB items to ensure consistency. Interviews were carried out in a private space within participants’ homes to reduce social desirability bias.

The study was approved by the Institutional Ethics Committee of Amrita School of Medicine, Kochi (ECASM-AIMS-2023-327). Written informed consent was obtained from all participants. Participation was voluntary, and confidentiality and anonymity were strictly maintained.

The primary outcomes were OHK and OHSB, classified as adequate or inadequate using the scoring method described above. Independent variables comprised sociodemographic characteristics, such as age, sex, religion, marital status, education, occupation, socioeconomic status, and family type, all collected as part of the structured questionnaire.

Data were entered into MS Excel (Microsoft Corporation, Redmond, Washington, United States) and analyzed using IBM SPSS Statistics for Windows, Version 21 (Released 2012; IBM Corp., Armonk, New York, United States). Categorical variables were summarized as frequencies and percentages, and continuous variables as means with standard deviations. Associations between OHK, OHSB, and sociodemographic variables were examined using chi-square tests. Variables with p < 0.20 in unadjusted analyses were included in multivariable logistic regression models to estimate effect sizes. As logistic regression was used, the results are presented as odds ratios (ORs) with 95% confidence intervals. There were no missing data, as the interviewer verified the completeness of all questionnaire responses during data collection. A p-value < 0.05 was considered statistically significant.

## Results

A total of 160 adults participated in the study. Participants’ ages ranged from 18 to 60 years, with a mean of 43.3 years (SD = 12.0). More than half were female (56.25%). The detailed sociodemographic profile is provided in Table 1.

**Table 1:** Sociodemographic characteristics of the respondents (N = 160) Median age = 45 years; ^i^ marital status based on the National Family Health Survey (NFHS) classification: widowed n = 10, separated n = 0, deserted n = 0, never married n = 21, divorced n = 0; ^ii^ less than primary schooling n = 13, primary schooling to higher secondary n = 80; ^iii^ homemaker n = 46, unemployed n = 8, student n = 8; ^iv ^based on the classification of socioeconomic status according to the ration cards provided by the government of India; ^v ^based on the classification of family types according to Brainly

Variables	n (%)
Age in years	
>=45	83 (51.87)
<45	77 (48.12)
Sex	
Female	90 (56.25)
Male	70 (43.75)
Religion	
Hindu	91 (56.87)
Christian	69 (43.12)
Marital status	
Currently married	129 (80.62)
Others ^i^	31 (19.37)
Education	
College education and above	83 (51.87)
Others ^ii^	77 (48.12)
Occupation	
Currently working	98 (61.25)
Others ^iii^	62 (38.75)
Socioeconomic status ^iv^	
Above Poverty Line	89 (55.62)
Below Poverty Line	71 (44.37)
Family type ^v^	
Joint family	80 (50.00)
Nuclear family	80 (50.00)

The prevalence of adequate OHK was 48.12% (95% CI: 38.58-57.80), while positive OHSB was observed in 41.25% (95% CI: 32.15-51.10). Multivariable analysis revealed that participants aged <45 years were more likely to have adequate OHK (AOR = 1.46; 95% CI: 1.15-2.12). Higher education (college and above) had a strong positive association with OHK (AOR = 5.31; 95% CI: 3.97-6.66). Being currently employed also showed a positive association (AOR = 1.69; 95% CI: 0.97-2.40). Socioeconomic status was an important determinant: individuals above the poverty line (APL) were more likely to report adequate OHK compared with those below the poverty line (AOR = 3.30; 95% CI: 2.41-4.19) (Table 2).

**Table 2:** Factors associated with oral health knowledge: results of multivariable analysis (logistic regression) (N = 160) COR: crude odds ratio; AOR: adjusted odds ratio; CI: confidence interval Bold values indicate statistical significance at p < 0.05, and variables that had a p-value < 0.2 in the unadjusted analysis were included in the adjusted analysis. Unadjusted analysis done using chi-square tests. Adjusted analysis done using multivariable logistic regression and are presented as odds ratios (OR) with 95% confidence intervals

Variable	n	Adequate n (%)	COR (95%CI)	AOR (95%CI)	p-value
Age in years					
<45	77	51 (66.23)	4.30 (2.22-8.34)	1.46 (1.15-2.12)	<0.001
>=45	83	26 (31.32)	Reference	Reference	-
Sex					
Female	90	46 (51.11)	1.32 (0.70-2.46)	-	0.391
Male	70	31 (44.28)	Reference	Reference	-
Religion					
Christian	69	34 (49.27)	1.01 (0.95-1.07)	-	0.816
Hindu	91	43 (47.25)	Reference	Reference	-
Marital status					
Currently married	129	65 (50.38)	1.61 (0.72-3.58)	0.98 (0.70-1.39)	0.930
Others	31	12 (38.71)	Reference	Reference	-
Education					
College education and above	83	74 (89.15)	202.82 (52.80-779.05)	5.31 (3.97-6.66)	<0.001
Others	77	3 (3.89)	Reference	Reference	-
Occupation					
Currently working	98	62 (63.26)	5.40 (2.65-10.99)	1.69 (0.97-2.40)	<0.001
Others	62	15 (24.19)	Reference	Reference	-
Socioeconomic status					
Above poverty line	89	69 (77.52)	27.17 (11.18-66.04)	3.30 (2.41-4.19)	<0.001
Below poverty line	71	8 (11.26)	Reference	Reference	-
Type of family					
Joint family	80	46 (57.50)	2.14 (1.14-4.02)	0.76 (0.13-1.39)	0.180
Nuclear family	80	31 (38.75)	Reference	Reference	-

Younger age (<45 years) was associated with more positive OHSB (AOR = 1.27; 95% CI: 1.04-1.54). Higher education (college and above) was a strong predictor (AOR = 4.84; 95% CI: 3.34-6.33). Socioeconomic status remained significant, with APL individuals more likely to demonstrate positive OHSB (AOR = 2.34; 95% CI: 1.54-3.13). Importantly, participants with adequate OHK were more than three times as likely to report positive OHSB compared with those with inadequate OHK (AOR = 3.35; 95% CI: 2.46-4.25) (Table 3).

**Table 3:** Factors associated with oral health-seeking behavior: results of multivariable analysis (logistic regression) (N = 160) COR: crude odds ratio; AOR: adjusted odds ratio; CI: confidence interval Bold values indicate statistical significance at p < 0.05; variables that had a p-value <0.2 in the unadjusted analysis were included in the adjusted analysis. Unadjusted analysis was done using chi-square tests. Adjusted analysis was done using multivariable logistic regression and is presented as odds ratios (OR) with 95% confidence intervals

Variable	n	Positive n (%)	COR (95%CI)	AOR (95%CI)	P value
Age in years					
<45	77	44 (57.14)	2.77 (1.70-4.51)	1.27 (1.04-1.54)	<0.001
>=45	83	22 (26.50)	Reference	Reference	-
Sex					
Female	90	39 (43.33)	1.31 (0.86-1.98)	1.22 (0.64-2.30)	0.544
Male	70	27 (38.57)	Reference	Reference	-
Religion					
Christian	69	33 (47.82)	1.61 (0.85-3.05)	-	0.718
Hindu	91	33 (36.26)	Reference	Reference	-
Marital status					
Currently married	129	53 (41.08)	1.43 (1.01-2.04)	0.97 (0.44-2.14)	0.931
Others	31	13 (41.93)	Reference	Reference	-
Education					
College education and above	83	64 (77.10)	126.32 (28.33-563.12)	4.84 (3.34-6.33)	<0.001
Others	77	2 (2.59)	Reference	Reference	-
Occupation					
Currently working	98	54 (55.10)	5.11 (2.43-10.77)	1.63 (0.89-2.38)	0.313
Others	62	12 (19.35)	Reference	Reference	-
Socioeconomic status					
Above poverty line	89	56 (62.92)	10.35 (4.67-22.92)	2.34 (1.54-3.13)	0.016
Below poverty line	71	10 (14.08)	Reference	Reference	-
Type of family					
Joint family	80	34 (42.50)	1.11 (0.59-2.08)	-	0.748
Nuclear family	80	32 (40.00)	Reference	Reference	-
Oral health knowledge					
Adequate	77	58 (75.32)	28.62 (11.70-69.99)	3.35 (2.46-4.25)	<0.001
Inadequate	83	8 (9.63)	Reference	Reference	-

The most frequently cited reason for visiting a dentist in the past year was tooth pain (61.25%). Conversely, the most common reason for avoiding dental care was the belief that minor dental problems could be self-managed (72.94%). Other reported barriers included lack of time (10.58%), absence of perceived need (5.88%), financial concerns (3.52%), accessibility issues (3.52%), and concurrent health problems (3.52%). Most participants (nearly 90%) sought care at private dental facilities, with only a minority utilizing government services. The main reason for preferring private facilities was the long distance to government dental centers (49.06%) (Table 4).

**Table 4:** Other factors associated with oral health-seeking behavior ^i ^decayed tooth n = 3, sensitivity n = 3, bleeding gums n = 1, orthodontic treatment n = 1;^ ii ^lack of time n = 9, no dental problems n = 5, financial instability n = 3, long distance n = 3, general health problems n = 3; ^iii^ private dental clinic n = 127, private dental college n = 6; * self-treatment/no dental treatment n = 15; ^iv^ don’t know where is the nearest government facility n = 7, not interested n = 3, quality of services are bad n = 3

Variables	n (%)
Reasons to visit the dentist in the last year (multiple responses (no: of participants = 75; no: of responses = 80))	
Tooth pain	49 (61.25)
Routine visit	23 (28.75)
Others^i^	8 (10.00)
Reasons for not visiting the dentist in the last year (N = 85)	
Mild dental issues that can be self-managed	62 (72.94)
Others^ii^	23 (27.05)
Type of dental institution where the participants underwent treatment (N = 145)	
Private Institution ^iii^	133 (91.72)
Government Institution	12 (8.27)
Reasons for not utilizing government dental services (multiple responses, number of participants = 145, number of responses = 161))	
Long distance	79 (49.06)
Fewer services	23 (14.28)
Long waiting time	20 (12.42)
Fewer staff	12 (7.45)
Others ^iv^	13 (.8.07)

Data on the distribution of responses to OHK and OHSB items are presented in Tables 5-6 (Appendices).

## Discussion

This community-based cross-sectional study assessed OHK and OHSB among rural adults in Ernakulam, Kerala, and identified key sociodemographic determinants. Less than half of participants demonstrated adequate OHK (48.12%) or positive OHSB (41.25%), underscoring considerable gaps in oral health literacy and utilization of care.

The proportion of adults with adequate OHK in this study is comparable to findings from Ghaziabad (50%) [2], but substantially lower than reported in Chennai (97.8%) [8]. Knowledge gaps were particularly evident in critical areas: only 40% knew how to select a toothbrush, 46.3% were aware of recommended replacement frequency, 15% recognized the importance of fluoridated toothpaste, and 38.8% were familiar with dental floss use. Awareness of the oral-systemic health link (46.3%) mirrored findings from one study [22], but differed from others [23]. Similarly, awareness of the importance of regular dental check-ups (45%) was consistent with an earlier report (44.4%) [4]. These deficits highlight the urgent need to strengthen oral health literacy in rural Kerala.

Positive OHSB was observed in only 41.25% of participants, aligning with studies from rural India [7,24,25], though higher than findings from Maharashtra [26] and lower than reports from Western populations, where up to 80% attend annual check-ups [27]. Notably, 9.4% had never visited a dentist, and only 14.4% attended six-monthly preventive check-ups, similar to a previous Indian study [26,28]. Consistent with earlier findings [7,28], tooth pain was the predominant reason for care-seeking, indicating reliance on symptomatic rather than preventive visits.

Decision-making was largely autonomous, with over 80% of participants independently choosing to seek dental care, echoing prior evidence [9]. However, some women, particularly those without independent income, depended on family members’ decisions, reflecting financial and gender-related constraints.

The most common reason for avoiding dental visits was the perception that minor dental problems could be self-managed (72.9%). This aligns with findings from other Indian studies [7], though it contrasts with reports where cost was the dominant barrier [26]. Other barriers included lack of time, financial concerns, and accessibility issues. Strikingly, nearly 90% of participants relied on private dental clinics, regardless of socioeconomic status, a pattern also observed elsewhere in India [23]. Limited availability and distance to government facilities (49.1%) were the primary deterrents, whereas other studies have reported affordability and perceived quality as decisive factors [7].

Younger participants (≤45 years) had significantly higher OHK and OHSB, consistent with some studies [29] but not others [26]. Younger adults may be more receptive to health messages, influenced by digital platforms, and more adaptable in adopting preventive practices [30]. Higher education emerged as a strong determinant of both OHK and OHSB, in line with previous findings [8,30]. Education likely enhances comprehension of preventive strategies and fosters critical health decision-making. Employment status also influenced OHK, possibly through greater exposure to structured routines, resources, and workplace health programs.

Socioeconomic status was a key determinant, with individuals APL demonstrating better outcomes in OHK and OHSB. This aligns with earlier evidence [2,31] that reflects better access to education and healthcare resources. Importantly, adequate OHK independently predicted positive OHSB, reinforcing the role of knowledge as a critical driver of oral health practices [16].

The findings highlight a dual challenge: limited oral health literacy and inadequate access to affordable public services. In rural Kerala, where private clinics dominate service delivery, strengthening government dental facilities is essential to reduce inequities. Community-based oral health education delivered through trusted platforms such as Anganwadi centres and primary care networks can bridge knowledge gaps and foster preventive practices. Integrating oral health into broader noncommunicable disease programs may also address common risk factors and maximize resource efficiency.

Strengths of this study include the use of a multistage cluster sampling method, inclusion of a broad adult age range, and comprehensive assessment of both OHK and OHSB using a validated tool. The high response rate (93%) adds to its robustness. The single-site design limits external validity, even though the selected Panchayat closely resembles other rural Panchayats. The cross-sectional design prevents causal inference, and reliance on self-reported data may have introduced recall or social desirability bias. Interviews conducted by a single investigator may also have led to interviewer bias, and the absence of clinical examinations limited verification of self-reported behaviours.

Recommendations

Strengthening public dental services is essential to improve oral health outcomes, particularly in rural areas. Expanding government dental facilities and enhancing accessibility can help reduce the population’s dependence on private clinics. Integrating oral health education into primary care and community platforms such as Anganwadi Centers, Accredited Social Health Activists (ASHAs), and self-help groups can further promote preventive care and encourage timely treatment-seeking behavior. Developing community-based oral health literacy programs is also crucial to address misconceptions, highlight the connection between oral and systemic health, and motivate individuals to adopt regular dental visits. Special attention should be given to high-risk groups, including older adults, individuals with lower educational levels, and socioeconomically disadvantaged populations, through tailored interventions aimed at reducing disparities. Moreover, incorporating oral health into broader noncommunicable disease (NCD) initiatives can leverage shared risk factors, thereby achieving a more cost-effective and sustainable public health impact. By addressing both knowledge and service gaps, policymakers and health systems can move toward equitable oral health outcomes. Future studies should consider multisite designs with clinical assessments to validate self-reported data and explore the effectiveness of community-based interventions in improving oral health literacy and behaviors.

## Conclusions

This study revealed that less than half of rural adults in Ernakulam district possessed adequate OHK (48.12%) or demonstrated positive OHSB (41.25%). Younger age, higher education, employment, and better socioeconomic status were significantly associated with improved OHK, while younger age, higher education, socioeconomic advantage, and adequate OHK predicted positive OHSB. Importantly, OHK emerged as a strong independent determinant of HSB. The findings highlight the prevailing reliance on symptomatic care; most visits were prompted by tooth pain, while preventive practices such as regular check-ups were uncommon. Barriers, such as perceptions of self-manageability of dental problems, poor accessibility of government facilities, and reliance on private care, emphasize the systemic and behavioral gaps in oral healthcare delivery.

## Appendices

**Table 5:** Oral health knowledge assessment: distribution of correct responses among participants (N = 160)

Variables	Correct response (n (%))
How many permanent teeth should be present in an adult’s mouth?	119 (74.37)
How frequently should one brush their teeth?	152 (95.00)
Appropriate method of brushing teeth?	76 (47.50)
Appropriate time spent on brushing teeth?	63(39.37)
Appropriate oral hygiene aid?	160 (100.00)
What is the preferred type of toothbrush, unless a dentist prescribes a specific one?	64 (40.00)
How often should a toothbrush be changed?	74 (46.25)
Appropriate cleaning material for teeth?	158 (98.75)
How do you select your toothpaste?	24 (15.00)
How much toothpaste should we use at a time for brushing teeth?	79 (49.37)
Have you heard about dental floss?	62 (38.75)
Purpose of dental floss?	42 (26.25)
When should dental flossing begin?	25 (15.62)
What are the reasons for brushing teeth?	79 (49.37)
Is tongue cleaning mandatory?	129 (80.62)
Does vigorous tongue cleaning cause loss of taste buds?	76 (47.50)
Have you heard about mouthwash?	135 (84.37)
When should one use mouthwash?	60 (37.50)
Does oral disease influence systemic health?	74 (46.25)
Can the avulsed tooth be re-implanted into the tooth socket, if immediate dental care by expertise is sought?	58 (36.25)
Should milk teeth require good care, even though they eventually fall out?	79 (49.37)
Does smoking/tobacco chew cause mouth diseases?	155 (96.87)
Does coffee and tea consumption cause staining of teeth?	88 (55.00)
Is bleeding from the gums while brushing teeth always normal?	74 (45.00)
Is there any negative link between frequent sugar consumption and dental caries?	147 (91.87)
Can irregularly placed teeth be corrected by an expert?	156 (97.50)
Is the condition of the teeth influenced by self-care rather than being determined at birth?	151 (94.37)
If gums are bleeding continuously, what should one do?	104 (65.00)
Does scaling harm the gums?	83 (51.87)
What should you do, if you come across initial caries (black dots) in any of your teeth?	64 (40.00)
A regular dental checkup must be in?	72 (45.00)
Is oral health an integral part of general health?	74 (46.25)
Do parafunctional habits such as thumb sucking and mouth breathing cause any abnormalities in orofacial structures?	83 (51.87)
Can parafunctional habits such as thumb sucking and mouth breathing be treated?	79 (49.37)
Have you heard about dentures?	157 (98.12)
How often should the denture wearer clean the denture?	82 (51.25)
What items should a denture wearer use for cleaning the denture?	66 (41.25)

**Table 6:** Oral health-seeking behavior assessment: distribution of positive responses among participants (N = 160)

Variables	Positive response (n (%))
Do you adhere to the regular oral hygiene measures (brushing frequency, technique, time of changing brush, etc)?	77 (48.12)
Do you consider dental treatment as important as other treatments?	57 (35.62)
Do you follow routine visits (every 6 months) to the dentist?	23 (14.37)
Have you visited a dentist at least once in the last year?	75 (46.87)
Decision maker regarding your dental healthcare-seeking?	139 (86.87)
Did you seek professional dental treatment at the earliest stage, rather than trying home remedies/self-treatment/taking advice from non-dental sources ever?	59 (36.87)
Did you miss any dental appointments after the initial visit/skip visit to the dentist despite experiencing dental problems more frequently?	60 (37.50)
